# Stereotactic Radiosurgery for Renal Cancer Brain Metastasis: Prognostic Factors and the Role of Whole-Brain Radiation and Surgical Resection

**DOI:** 10.1155/2015/636918

**Published:** 2015-11-19

**Authors:** Franziska M. Ippen, Anand Mahadevan, Eric T. Wong, Erik J. Uhlmann, Soma Sengupta, Ekkehard M. Kasper

**Affiliations:** ^1^Department of Neurosurgery, Beth Israel Deaconess Medical Center, Harvard Medical School, Boston, MA 02445, USA; ^2^Department of Radiation Oncology, Beth Israel Deaconess Medical Center, Harvard Medical School, Boston, MA 02445, USA; ^3^Department of Neuro-Oncology, Beth Israel Deaconess Medical Center, Harvard Medical School, Boston, MA 02445, USA

## Abstract

*Background*. Renal cell carcinoma is a frequent source of brain metastasis. We present our consecutive series of patients treated with Stereotactic Radiosurgery (SRS) and analyse prognostic factors and the interplay of WBRT and surgical resection.* Methods*. This is a retrospective study of 66 patients with 207 lesions treated with the Cyberknife radiosurgery system in our institution. The patients were followed up with imaging and clinical examination 1 month and 2-3 months thereafter for the brain metastasis. Patient, treatment, and outcomes characteristics were analysed.* Results*. 51 male (77.3%) and 15 female (22.7%) patients, with a mean age of 58.9 years (range of 31–85 years) and a median Karnofsky Performance Status (KPS) of 90 (range of 60–100), were included in the study. The overall survival was 13.9 months, 21.9 months, and 5.9 months for the patients treated with SRS only, additional surgery, and WBRT, respectively. The actuarial 1-year Local Control rates were 84%, 94%, and 88% for SRS only, for surgery and SRS, and for WBRT and additional SRS, respectively.* Conclusions*. Stereotactic radiosurgery is a safe and effective treatment option in patients with brain metastases from RCC. In case of a limited number of brain metastases, surgery and SRS might be appropriate.

## 1. Background

Renal cell carcinoma (RCC) accounts for about 2% of all cancer cases worldwide and represents the sixth leading cause of all cancer deaths [1, 2]. One-third of patients present at advanced stages of disease, and up to 40% of patients who underwent local surgical resection will have disease recurrence [3, 4].

Despite its relatively low incidence, RCC presents itself as one of the most common sources of brain metastases along with lung and breast cancer, melanoma, and colorectal carcinoma [5]. Approximately 1,200 to 1,500 cases of brain metastases from RCC are diagnosed annually [6], and 4% to 17% of all patients with RCC will develop brain metastases during their clinical course of disease [7].

The median survival of patients with untreated brain metastases from primary RCC is reported to be approximately 1 to 2 months [7], whereas the median survival time after radiotherapy and corticosteroid treatment for patients with this type of malignancy was reported to be 2 to 8 months [8]. Since surgical resection is not always possible, WBRT has played an important role in the treatment of patients with RCC brain metastasis but has yielded unsatisfactory results in terms of overall survival and local tumor control in these patients due to the relative radioresistant nature of RCC to conventional radiation therapy [9]. Due to the potential neurotoxic effects of WBRT as well as the radioresistant features of this primary, WBRT may not be the treatment of choice in these patients, particularly with oligometastatic disease [10].

Stereotactic radiosurgery (SRS) is a minimally invasive radiation technique that delivers a highly conformal, high dose of radiation to a prescribed target volume [11, 12]. This procedure can be completed in one up to five treatment sessions and offers the possibility to treat multiple tumor sites during one treatment session [12]. Stereotactic radiosurgery is increasingly used for the treatment of brain metastases with or without prior microsurgical resection [13, 14], as tumors traditionally considered to be radioresistant such as renal cell carcinoma have shown favorable response rates in various studies [7, 15–20]. However, the optimal treatment of these patients still remains controversial.

In this study, our aim was to analyze the outcomes after SRS for the treatment of brain metastases from RCC. Furthermore, we examined potential prognostic factors that correlate with improved survival and local tumor control in these patients.

## 2. Methods

### 2.1. Study Design

This is a retrospective evaluation of all patients treated with SRS for brain metastases from primary RCC at our institution. Patients' medical records were reviewed to obtain patient, tumor, and treatment characteristics and follow-up data. Neuroimaging studies for each individually treated lesion were reviewed prior to radiosurgical treatment and at regular intervals (1, 3, 6, 9, 12, and 24 months) after completion of SRS. Data were collected by personnel not directly involved in either direct patient care or any related treatment decision-making process. The design and analysis of this study were approved by the Institutional Review Board (IRB) of Dana Farber/Harvard Cancer Center (DF/HCC) (IRB#09-451).

### 2.2. Patient Selection

The study cohort consists of 76 patients with RCC brain metastases treated with SRS at BIDMC between August 2005 and December 2013. For 10 (13.2%) patients, no follow-up was available because they transferred their care to other facilities. These patients were excluded from any further analysis. Evaluation of overall survival, local and distant brain tumor control were performed for the remaining 66 patients with a total of 207 lesions, for which all follow-up data sets were available and analysis was completed.

In 65/66 patients (98.5%), brain metastases from RCC were diagnosed by magnetic resonance imaging (MRI). In one patient (1.5%), the diagnosis was based on computed tomography (CT) alone, since the patient harbored a contraindication to undergo MRI scanning. Each patient's performance status was assessed at each visit using the Karnofsky Performance Status (KPS) and was further classified by prognosticators for assessment of their outcomes.

### 2.3. SRS Planning and Treatment

All patients were treated in the Cyberknife (Accuray Inc., Sunnyvale, California) robotic frameless stereotactic radiosurgery system. Diagnostic thin slice (1 mm) gadolinium enhanced axial MRI images were fused with CT scan obtained in an immobilization mask at planning. Image fusion and nonisocentric treatment planning were performed with the multiplan treatment planning software.

### 2.4. Follow-Up

Patients were followed up from the time of SRS with clinical examination and neuroimaging with contrast enhanced MRI 1 month after treatment and every 2-3 months thereafter until the last follow-up appointment or until the date of death.

### 2.5. Outcome Measures

Overall survival, local control, distant brain control, local progression-free survival, and distant brain progression-free survival were assessed after SRS. Overall survival was calculated as the time in months from SRS until the date of death. In case of censored data, the patients' last date of clinical follow-up visit was used to determine overall survival.

A determination of the cause of death was attempted for all patients who died during the observation period. Patients were considered to have died due to neurologic causes if they had either absent or stable systemic disease and progressive neurologic dysfunction. If patients had developed fatal organ failure, infection, or hemorrhage, in the setting of a stable neurological examination at the last clinic visit, they were considered to have died from progression of systemic disease or intercurrent disease (e.g., pulmonary embolus) and not due to neurological causes.

Treatment response was evaluated according to the updated Response Evaluation Criteria in Solid Tumors (RECIST). Local control (LC) was defined as no further tumor growth after treatment, subdivided into complete response (CR), partial response (PR), or stable disease (SD) on follow-up CT and/or MRI scans. In lesions which underwent resection prior to SRS, LC was defined as the absence of new nodular contrast enhancement adjacent to the resection cavity on MRI. Local failure (LF) was defined as tumor recurrence at the site of the targeted lesion and was further classified as progressive disease (PD). Distant brain control (DC) was defined as the absence of new intracranial lesions after treatment, whereas distant brain failure (DF) was defined by the appearance of new brain metastases or leptomeningeal disease outside the lesions previously treated with SRS. Actuarial local progression-free survival (LPFS) and distant brain progression-free survival (DPFS) were calculated in months from the date of SRS to the date of CT/MR-imaging showing local or distant brain failure. Otherwise, patients were censored at the time of their last MRI scan. For patients receiving WBRT for salvage, control rates were censored at the time of WBRT.

### 2.6. Statistical Analysis

Descriptive statistics were obtained for a variety of patient and treatment characteristics in this study. Actuarial OS, LC, and DF rates were calculated using the Kaplan-Meier method. Univariate analysis was performed using the log-rank test for categorical data. Multivariate analysis was performed using Cox proportional hazards regression for continuous variables and in order to identify prognostic factors for OS and LPFS. For both univariate and multivariate analyses, statistical significance was defined as a level of *α* = 0.05 value.

Kaplan-Meier curves for OS, LPFS, and DPFS and univariate analysis were conducted using Graph Pad Prism version 6.00 software for Mac (Graph Pad Software, San Diego, CA; Windows; Microsoft, Seattle, WA). Descriptive statistics and multivariate analyses were performed using the STATA 13 software package (STATA Corp., College Station, TX, USA).

## 3. Results

In a total of 66 patients with 207 brain metastases, the median follow-up after SRS was 10 months (mean, 15.8 months; range, 6–84 months).

### 3.1. Patient Characteristics

Of the analyzed 66 patients, 51 were male (77.3%) and 15 were female (22.7%). The patients ranged in age from 31 to 85 years (mean age of 58.9 years) at the time of their initial brain metastasis diagnosis. The median Karnofsky Performance Status (KPS) was 90 (range of 60–100). Thirty-nine patients (59.1%) presented with a single brain metastasis; 27 patients (40.9%) had two or more brain metastases at time of diagnosis. At the time of SRS treatments, 56 patients (84.8%) were found to have uncontrolled systemic disease and 10 patients (15.2%) were found to have controlled systemic disease. According to the Recursive Partitioning Analysis (RPA) by the Radiation Therapy Oncology Group (RTOG), 3 patients (4.5%) were classified as RPA class I, 59 patients (89.4%) as RPA class II, and 4 patients (6.1%) as RPA class III. Patients were also classified into subgroups according to the Score Index for Radiosurgery (SIR) and the Basic Score for Brain Metastases (BSBM) to allow a prognostic determination of patients with brain metastasis who underwent SRS and to make this data set comparable to other available literature. According to the SIR, 35 patients (53%) were found to have a score less than 6, and 31 patients (47%) were found to have a score equal to and more than 6. According to the BSBM, 12 patients (18.2%) had a score of 0, whereas 44 patients (66.7%) had a score of 1 and 8 patients (12.1%) were found to have a score of 2. Two patients (3%) had a score of 3. According to the Disease-Specific Graded Prognostic Assessment (Ds-GPA), 27 patients (40.9%) were classified as Ds-GPA 4, 18 (27.3%) as Ds-GPA 3, 14 (21.2%) as Ds-GPA 2, 6 (9.1%) as Ds-GPA 1, and 1 (1.5%) as Ds-GPA 0.

In 51 patients (77.3%), the histologic subtype was defined as clear cell carcinoma, but also two cases of papillary RCC (4.5%) and one case of chromophobe RCC (1.5%) were observed. In 12 patients (18.2%) with brain metastases from RCC, the histologic subtype remained unclassified.

At the time of diagnosis of the first brain metastasis, 63 patients (95.5%) also had extra cranial metastases. Patient and disease characteristics are shown in Table 1.

### 3.2. Treatment Characteristics

Stereotactic radiosurgery with the Cyberknife (Accuray, Sunnyvale, CA) technique was used to treat all patients in this cohort with brain metastases from RCC. A total of 207 lesions were treated in 179 separate sessions via a total of 132 treatment plans. An average of 1.2 lesions was irradiated in each treatment session and an average of 1.6 lesions was irradiated in each treatment plan (range, 1–6).

The median prescription dose was 22 Gy, the median conformality index was 1.3 (range of 1.03–6.96), and the median homogeneity index was 1.32 (range, 1.12–1.72). The median prescribed isodose line was 76% (range of 58–89%) and the median coverage of each individual lesion was 96.39% (range, 84.2%–100%). All patients received prophylactic corticosteroids (dexamethasone) and anticonvulsants (levetiracetam) during and after the SRS treatment.

56 patients (84.8%) received additional systemic therapy during their course of disease. Of those, 25 patients (44.6%) received standard systemic therapy (e.g., high dose IL2; sunitinib and pazopanib), 5 patients (9%) were treated with IRB-approved experimental study therapy regimens (e.g. bevacizumab + interferon *α*; bevacizumab versus erlotinib; and pazopanib versus sunitinib, or tivozanib), and 26 patients (46.4%) were treated with a combination of both. 10 patients (15.2%) had received no systemic therapy at all at the time of SRS treatment.

As an initial treatment, 24 patients (36.4%) underwent surgical resection before SRS, 36 patients (54.5%) were treated with SRS only, and 6 patients (9.1%) had received prior WBRT (median dose, 30 Gy; range, 20–30 Gy). Six patients (9.1%) were treated with WBRT for salvage after SRS and 2 patients (3%) had resection due to progression after treatment with SRS. In patients initially treated with surgical resection prior to SRS, gross total resection could be achieved in 22 patients (91.7%). Treatment characteristics are shown in Table 2.

### 3.3. Overall Survival

**Figure 1:**
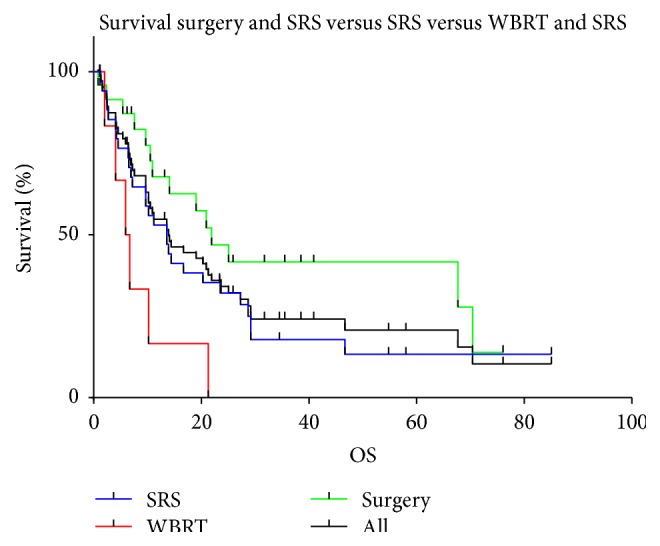
Overall survival by treatment modality.

**Figure 2:**
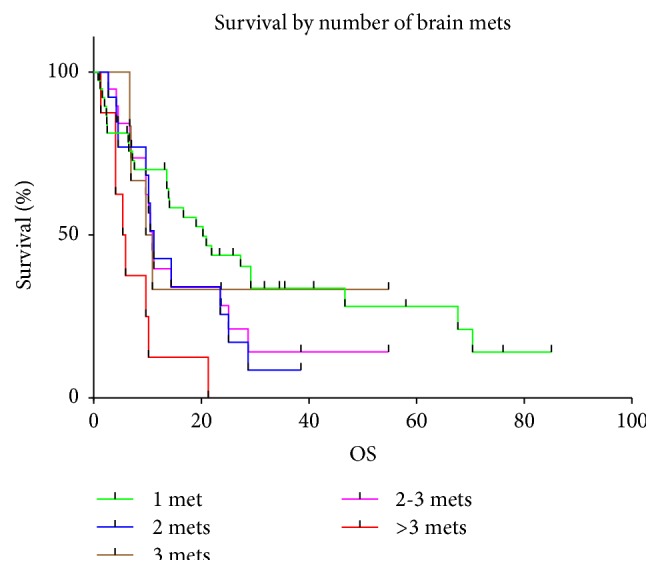
Overall survival by number of brain metastases.

**Figure 3:**
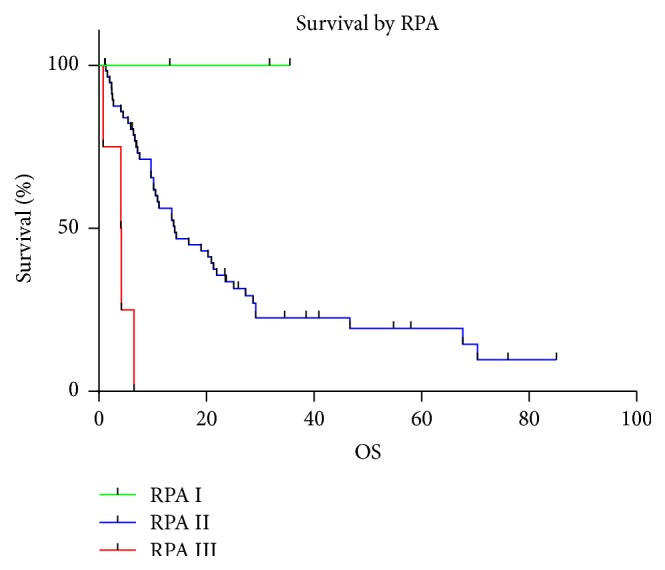
Overall survival by RPA (Recursive Partitioning Analysis) class.

At the time of analysis (6 months after the last SRS treatment), 48 patients were dead (72.7%) and 18 were alive (27.3%). Most of the deceased patients (24; 50%) died from documented progression of systemic disease (nonneurological death), whereas in 21 patients (43.75%), the specific cause of death was unknown in the setting of a stable neurological examination at last visit and 3 patients (6.25%) died from progression of intracranial disease (neurological death). The median overall survival was 72.2 months (95% CI 45.2–95.5 months) from the diagnosis of the primary tumor, 17.5 months (95% CI 11.5–22.5 months) from the diagnosis of the first brain metastasis, and 13.9 months (95% CI 9.7–21.3 months) from the time of SRS for the analyzed study population. Actuarial survival rates for the analyzed patient cohort calculated from the time of SRS were 98.5% (*n* = 65) at 1 month, 87.4% (*n* = 55) at 3 months, 77.8% (*n* = 49) at 6 months, 68% (*n* = 41) at 9 months, 54.8% (*n* = 33) at 12 months, and 34.1% (*n* = 18) at 24 months (Figure 1). The median overall survival from the time of SRS for the 39 patients with a single brain metastasis was 20.3 months (95% CI 13.6–29.2 months) compared to 5.4 months (95% CI 1.3–10.2 months) in 8 patients with multiple (>3) brain metastases (*p* = 0.0022) (Figure 2). No statistically significant difference in median overall survival was found when comparing patients with a single brain metastasis to patients with two (11.2 months) or three brain metastases (9.7 months) at initial presentation (*p* = 0.1853). The median OS was significantly different for the three different RPA classes (*p* = 0.0001). In patients stratified into RPA class I, median OS was not reached because all 3 patients were still alive at the time of analysis. Patients in RPA class II and III only had a median survival of 14.1 months and 4.1 months respectively (Figure 3).

Stratifying the patients cohort by their initial treatment modality (surgery prior to SRS, SRS, and WBRT prior to SRS) resulted in a median survival for patients initially treated with SRS only (*n* = 36) of 13.6 months (95% CI 6.9–23.5 months) and a median survival of 21.9 months (95% CI 10.5–70.4 months) for patients who underwent surgical resection (*n* = 24) as an initial treatment. Patients who underwent WBRT (*n* = 6) before treatment with SRS had a median survival of 5.9 months after SRS (Figure 1). The actuarial one-year overall survival rates for patients treated with those different approaches were 55.9% for patients who underwent SRS as a sole treatment, 67.8% for patients who underwent surgical resection prior to SRS, and 16.7% for patients treated with WBRT prior to SRS (*p* = 0.011). No significant difference in overall survival was detected between patients treated with SRS only and patients treated with surgery + SRS (*p* = 0.1141).

In univariate analysis of the entire cohort age (*p* = 0.0000), prior surgery (*p* = 0.0486), RPA class (*p* = 0.0000), KPS (≥70 versus <70, *p* = 0.0000), SIR (≥6 versus <6, *p* = 0.0093), BSBM (*p* = 0.0027), number of brain metastases (>3 versus ≤3, *p* = 0.0009), initial tumor volume (*p* = 0.0000), and Ds-GPA (*p* = 0.0002) were associated with significantly better overall survival. Prior WBRT (*p* = 0.0097) was found to be significantly associated with poor overall survival.

Sex, systemic and intracranial disease status at the time of SRS, and whether the patients had received systemic treatment during their course of systemic disease were not found to be significantly associated with a difference in overall survival.

In multivariate Cox analysis, factors associated with a significantly better overall survival were age (*p* = 0.038), RPA class (*p* = 0.000), KPS (≥70 versus <70, *p* = 0.000), and the initial number of brain metastases (>3 versus ≤3, *p* = 0.002). Again, prior WBRT was significantly associated with poorer overall survival (*p* = 0.014). Prior surgery (*p* = 0.053) was only found to be borderline significant in multivariate Cox regression.

Factors not found to be significantly associated with better overall survival were sex, systemic and intracranial disease status at the time of SRS, whether the patients had received systemic treatment during their course of systemic disease, and initial tumor volume (Table 3).

In univariate analysis of the two subgroups initially treated with SRS and surgical resection followed by SRS, age (*p* = 0.0000), RPA class (*p* = 0.0001), KPS (≥70 versus <70, *p* = 0.0001), SIR (≥6 versus <6, *p* = 0.0385), BSBM (*p* = 0.0147), Ds-GPA (*p* = 0.0001), initial tumor volume (*p* = 0.0000), and the initial number of brain metastases (>3 versus ≤3, *p* = 0.0002) were found to have a significant impact on overall survival. In multivariate Cox analysis of these two subgroups, again RPA class (*p* = 0.000), KPS (≥70 versus <70, *p* = 0.001), SIR (≥6 versus <6, *p* = 0.043), Ds-GPA (*p* = 0.003), BSBM (*p* = 0.008), and the initial number of brain metastases (>3 versus ≤3, *p* = 0.001) were found to be prognostic for better overall survival. In multivariate analysis, the initial tumor volume was not found to be a prognostic factor for overall survival.

### 3.4. Local Control

**Figure 4:**
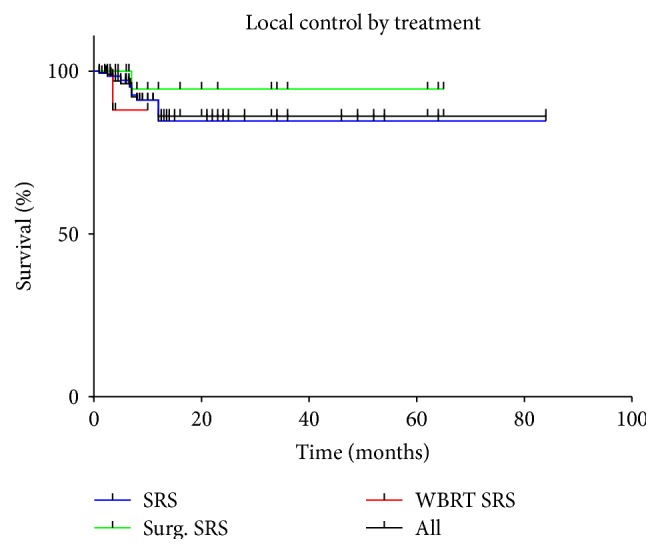
Local control by treatment modality.

Over the course of the entire follow-up period, local control was achieved in 193 (93.2%) of 207 treated lesions. Of the 138 lesions treated with SRS only in a total of 51 patients, local failure was noted in 10 lesions (7.2%) of 8 patients (15.7%) during the entire follow-up period. In a total of 25 lesions treated with surgery and SRS as an adjunct in 24 patients, local failure was noted in 1 lesion (4%) in 1 patient (4.2%). Of the 44 lesions treated with WBRT prior to SRS in a total of 6 patients, local failure was observed in 3 lesions (6.8%) in 1 patient (16.7%). Actuarial 1-year local control rates for lesions treated with SRS as a sole treatment, surgical resection + SRS, and WBRT + SRS were 84%, 94%, and 88%, respectively (Figure 4).

In univariate analysis, no significant difference in local control was found between lesions treated with the three different approaches (*p* = 0.445). Furthermore, no statistically significant difference in local control could be detected comparing SRS with surgical resection + SRS (*p* = 0.3422), SRS with WBRT + SRS (*p* = 0.445), and WBRT+ SRS with surgical resection + SRS (*p* = 0.333).

Tumor volume was found to be the only significant variable in univariate log-rank analysis (*p* = 0.0000); however, in multivariate Cox analysis, neither tumor volume nor surgical resection or the number of brain metastases was found to be prognostic for local progression-free survival.

### 3.5. Distant Brain Progression-Free Survival

**Figure 5:**
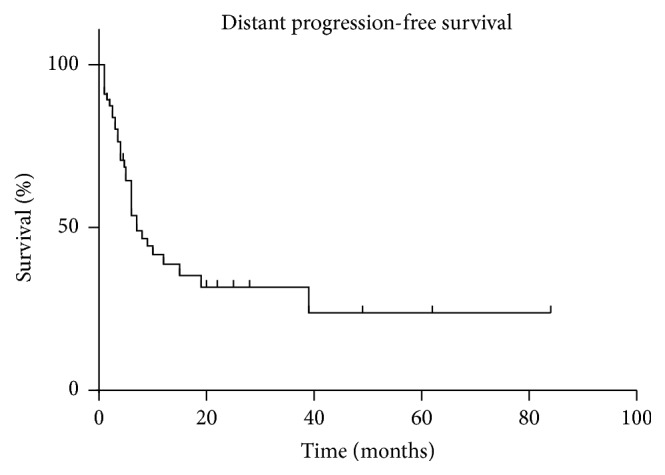
Distant brain progression-free survival.

Distant brain failure was observed in 34 (51.5%) patients. The median time until distant brain failure was 7 months after SRS (95% CI 6–15 months). Actuarial freedom from distant brain failure was 90.9% at 1 month, 76.3% at 3 months, 48.9% at 6 months, 41.7% at 9 months, 35.2% at 12 months, and 23.8% at 24 months after SRS. The distant brain progression-free survival for all patients is shown in Figure 5. Median distant brain progression-free survival for patients who initially received SRS alone, surgery + SRS, and WBRT + SRS was 19, 7, and 3.5 months, respectively.

In univariate as well as in multivariate analysis, prior WBRT was significantly associated with better distant tumor control (*p* = 0.007 in univariate analysis and *p* = 0.014 in multivariate analysis).

### 3.6. Complications after Treatment

14 (21.2%) patients developed some form of toxicity related to SRS. Of the 24 patients initially treated with surgical resection, 7 had side effects: among this group, 6 patients developed fatigue, one patient additionally experienced worsening of his left-sided weakness after SRS (1/24 acute grade 3 toxicity). Among 36 patients who received SRS only as an initial treatment, 7 patients had side effects. 5 patients developed fatigue, and among those, one patient had seizures due to expanding vasogenic edema after treatment. Of the remaining two patients, one patient experienced worsening edema causing mass effect and midline shift (2/24 Acute Grade 3 Toxicity); the other presented with symptomatic radiation necrosis causing left hemiplegia. In the group of patients who received upfront WBRT, one patient developed nausea after treatment with WBRT, before undergoing SRS. Overall, there were 4/66 (4.5%) acute grade ≥ 3 toxicity and 1/66 (1.5%) ≥ grade 3 long term toxicity. The mean volume in patients experiencing toxicity was 12.6 and the median dose was 20 Gy.

## 4. Discussion

Brain metastases from RCC are reported with a frequency of approximately 4 to 17% of patients during their course of disease [7]. As newer therapies for the management of RCC emerge and standard treatments are further refined, these patients will live longer and, as a consequence, are more likely to develop brain metastases during their course of disease [21]. In addition, brain metastases from RCC are known for their high propensity of intratumoral hemorrhage and their extensive surrounding edema which is profound when compared to other metastatic brain lesions from other primaries [9]. Surgery may often not be feasible due to location of the lesion and studies on WBRT as a sole treatment in the treatment process of these patients have shown disappointing results regarding overall survival [22–27]. SRS is proving to be a useful modality in the treatment of brain metastasis from RCC.

In this retrospective cohort analysis, we evaluated the effectiveness, safety, and potential prognostic factors of SRS for the treatment of brain metastases from RCC on survival and local and distant tumor control at our institution.

### 4.1. Stereotactic Radiosurgery and Overall Survival

Stereotactic radiosurgery for brain metastases from RCC has been suggested to prolong overall survival when compared to patients treated with modalities such as WBRT only [15, 20, 28] with median survival rates of 5.1 to 17.8 months. In our study, the median survival of the entire cohort was 13.9 months. The groups treated with SRS only, surgical resection plus subsequent SRS, and WBRT plus SRS achieved a median overall survival of 13.6, 21.9, and 5.9 months, respectively. The results for survival among the three groups were found to be statistically significant. A significant difference in median overall survival was furthermore observed when stratifying the patients into the three different RPA classes. This result confirms findings from previously published series evaluating the role of SRS for patients with brain metastases from this primary [21, 29, 30]. The majority of patients in our series were graded into RPA class II due to the presence of active extracranial disease or advanced age.

### 4.2. Stereotactic Radiosurgery and Local Tumor Control

Multiple studies have reported the effectiveness of SRS for brain metastases from RCC have reported local control rates ranging from 60.9 to 100% (Table 4) [7, 8, 14–16, 18–21, 24, 28–42]. The majority of patients in these studies were treated with LINAC-based or Gamma Knife-based devices, but comparable data on treatment outcomes for these patients undergoing Cyberknife SRS is lacking. To date, our retrospective study is the largest study conducted so far to evaluate the outcome of patients and potential prognostic factors in the treatment of Cyberknife radiosurgery for brain metastases from RCC.

In our study, actuarial 1-year local control rates for lesions treated with the three different initial treatment approaches were 84% for patients treated with SRS as a sole treatment, 94% for patients who underwent surgical resection plus subsequent SRS, and 88% for patients initially treated with WBRT followed by SRS which is comparable to that in literature. Except for a single report [48], SRS as a sole treatment or in combination with WBRT has shown favorable results comparable to those of surgery plus subsequent WBRT in patients with a single brain metastasis in the current literature [49–51].

Several important factors such as tumor size, location, number of brain metastases, presence of symptomatic peritumoral edema, and mass effect have to be taken into consideration when it comes to the decision whether a patient should undergo surgical resection or SRS. In regard to the tumor size, the RTOG protocol 90-05 established SRS dose-volume prescription criteria, recommending a maximum dose of 24, 18, and 15 Gy in a single fraction for tumors ranging up to 2 cm, between 2 and 3 cm, and greater than 3 cm in diameter, respectively [6, 10]. Other reported optimal doses range from 15 up to 22 Gy, with a median dose of 20 Gy [6].

The treatment of patients with brain metastases from RCC has several advantages: stereotactic radiosurgery is a minimally invasive procedure, usually performed in the outpatient setting, with the potential to treat multiple lesions during one treatment session, and can be performed repeatedly in case of local or distant brain tumor recurrence [12, 52]. However, despite the noted improvements in local tumor control, some questions still remain to be investigated in the field of this treatment modality, such as an appropriate selection of patients for SRS versus surgery, the development of validated prognostic factors after treatment with SRS, and the role of adjuvant WBRT [10]. The role of adjuvant treatment options such as WBRT and targeted systemic treatments especially needs to be further investigated in brain metastases from RCC.

### 4.3. Stereotactic Radiosurgery Related Toxicities

During the course of treatment, a total of 4 (6%) patients of the analyzed cohort presented with ≥ grade 3 side effects related to SRS. Especially among the patients with severe complications such as symptomatic radiation necrosis, worsening symptoms, and seizures related to the treated lesion, there was trend towards larger initial tumor volume. The more severe side effects were more common among the patients with larger lesions treated with SRS only than in the surgical group.

### 4.4. Surgery

In patients with a single brain metastasis, with good performance status, and limited to controlled systemic disease, surgical resection of brain metastasis has shown survival benefit in randomized data [53], but, however, there is currently no class I evidence available for the optimal surgical treatment of patients with 2 or more brain metastases [54]. Since local failure rates as high as 60% after surgical resection have been reported, adjuvant SRS or WBRT is recommended [54, 55]. Two randomized trials [53, 56] have furthermore shown that the addition of WBRT to either surgery or SRS results in significantly improved local and distant brain tumor control, although improved overall survival has only been reported for surgery plus subsequent WBRT [53].

In addition, Bindal and colleagues [57] reported equivalent survival time of patients with up to three brain metastases and good performance status who had all lesions removed to that of similar patients undergoing surgery for a single brain lesion.

To date, randomized trials reporting significantly improved survival, local and distant brain control after treatment with surgery, and/or SRS have only been including patients with brain metastases from different primary tumors, but no such trials have been conducted on the surgical treatment for patients with brain metastases from renal cell carcinoma [43].

In our study, the group of patients initially treated with surgical resection followed by SRS had a median survival of 21.9 months and the highest 1-year local tumor control rate (94%), although these results failed to show statistically significant difference compared to the outcome of patients initially treated with SRS only, which may be due to the relatively low number of patients in our cohort. This observation should hence be reexamined in a larger sample which can be achieved by pooling data or when patients are accrued in a multicenter trial.

Based on the available data in the current literature, the first-line treatment for accessible brain metastases from RCC has been surgical resection followed by WBRT. To date, six studies have been conducted to evaluate the outcome and prognostic factors of patients who underwent surgical resection for brain metastases from RCC [58–63].

In aggregate, the results of these investigations including our findings support the role of surgery for brain metastases from RCC in selected patients with good prognostic factors, limited or controlled systemic disease, and a single brain metastasis in a surgically accessible location, as surgery usually results in immediate relief of symptoms and can contribute to achieving excellent local tumor control. However, in terms of quality of life, even patients with poor prognostic factors may also benefit from surgical intervention if a lesion causing significant mass effect can be removed [64]. The question whether surgical resection only or a combined approach is more favorable still remains unclear as prospective randomized trials for patients with brain metastases from RCC are lacking.

### 4.5. WBRT

Historically, whole-brain radiation therapy (WBRT) has been the mainstay of treatment in the management of patients with brain metastases, although this treatment modality is potentially associated with neurocognitive dysfunction and with suboptimal control rates, especially for larger tumors [10, 52]. Outcomes after WBRT appear especially poor for patients with metastatic RCC, as this tumor has traditionally been considered to be relatively radioresistant compared to brain metastases from other primaries, such as lung or breast [9, 10].

In our study, patients treated with WBRT prior to SRS had the second highest local control rate. Furthermore, upfront WBRT was significantly associated with improved distant brain tumor control in univariate and multivariate analysis, although this treatment combination revealed a median survival of 5.9 months in these patients, a significantly worse result compared to the overall survival of patients initially treated with SRS only and patients treated with surgery and SRS as an adjunct. This could be attributed to selection bias.

In three retrospective studies, the outcomes of patients with brain metastasis from RCC treated with WBRT were evaluated: the first study conducted by Wroński et al. [27] revealed a median survival of 3.3 months calculated from the last day of WBRT, with death from neurologic causes in 76% of patients. One year later, another study to further investigate the question whether WBRT is a suitable treatment for patients with metastatic brain lesions from RCC was published by Culine et al. [23]. The median survival of patients who received radiotherapy alone was 7 months, compared to a median survival of 1 month of patients who did not undergo any specific treatment and 10 months of patients who underwent surgery. In 2004, Cannady and colleagues [22] published another study with comparable survival rates to those of Wroński et al., with a median survival after WBRT of 3.3 months in a total of 46 patients who received WBRT as their initial treatment for brain metastasis. Furthermore, the median survival rates for the different RPA classes were evaluated: the median survival for RPA classes I, II, and III was 8.5, 3, and 0.6 months, respectively, but no statistically significant difference was observed among the three classes [22].

In addition, some studies also revealed the potential benefit of dose escalation [22, 65–67]. The most recent study was conducted by Rades et al. [66], in which higher doses of radiation (40 Gy in 20 fractions or 45 Gy in 15 fractions) compared to standard treatment regimens resulted in improved local control and overall survival rates. Patients who were treated with higher doses had a median overall survival of 12 months and 6 months' local control rates of 57%, compared to patients treated with lower doses, who had a median overall survival of 4 months and 6 months' local control rates of 21% [66].

These rather unsatisfactory results of treatment or RCC metastasis with WBRT only led to the implementation of more aggressive treatment approaches for brain metastasis from RCC, such as surgical resection and SRS [10].

In 1987, Gay et al. [25] analyzed the median survival rates of 25 patients who received radiation therapy only (13 weeks) and 7 patients who underwent surgical resection and postoperative radiation (66 weeks). However, interpreting the results of this study, it has to be kept in mind that the patients who underwent surgery were preselected because of stable systemic disease, an accessible single metastatic lesion, and the belief that the tumor burden could be completely resected.

Ikushima et al. [26] extended the available data with their retrospective analysis of the effect of adjuvant fractionated stereotactic radiotherapy (FSRT) after surgery compared to surgical resection with adjuvant WBRT and WBRT alone. The different treatment groups achieved median survival times of 25.6, 18.7, and 4 months, respectively. The results in this study, however, are confounded by the fact that the patients included had a relatively good performance status compared to the patients of other studies conducted on this topic before and FRST was only indicated in patients with a good performance status and a tumor diameter of ≤3 cm and if patients presented with less or equal to 3 lesions [9].

A recent study published by Fokas and colleagues [24] evaluated the role of the treatment with SRS and WBRT in brain metastasis from RCC in a total of 88 patients. Fifty-one patients were treated with SRS, and 17 were treated with SRS plus adjuvant WBRT, whereas the remaining 20 patients were treated with WBRT only. The median overall survival for these different treatment groups was 12, 16, and 2 months, respectively. Statistically significant difference was found in overall survival rates of patients treated with SRS only as well as patients treated with a combination of SRS and WBRT compared to patients treated with WBRT only [24].

Taking everything into account, the results of these studies, including our retrospective analysis, suggest improved local and distant brain tumor control for brain metastases from RCC when WBRT is administered. Although RCC is considered to be a radioresistant tumor, these results suggest that there might be an effect of WBRT on microscopic metastases from RCC within the brain or a potential delay in the appearance of new brain metastases. However, no significant survival benefit could be demonstrated in these patients. This result might be partially explained by selection bias, because WBRT was more commonly used in patients with a larger number of brain metastases.

Our results suggest that more aggressive treatment options like surgical resection and SRS, possibly in combination with WBRT, might be beneficial for patients with favorable performance status and a limited number of brain lesions. However, WBRT and supportive care continue to be the treatment of choice in patients with multiple brain metastases, poor performance status, uncontrolled systemic disease, and a short life expectancy [52, 68]. As more aggressive treatment options may be associated with an increased risk in these patients, it is important to take into account the prognosis of each patient in order to individualize the treatment approach [69] and to offer the best possible treatment modality for an improved outcome of these patients.

### 4.6. Limitations of This Study

The present study has inherent limitations based on its retrospective nature, and the obtained results may be somewhat influenced by clinical selection bias. In light of varying treatment regimen during the course of disease of the analyzed patients, reliable prognostic factors remain difficult to assess. Furthermore, complete follow-up was only available for 86.8% of all patients. The other patients were transferred to other facilities for further follow-up and could not be analyzed in this study. Therefore, despite the fact that this cohort is the largest reported series to date, the analyzed cohort is a rather heterogeneous group of patients with a variety of different systemic treatment regimens, prior WBRT, and prior surgery, or patients treated with SRS only. Due to this fact, it is difficult to analyze the exact impact of the different treatment options as well as potential prognostic factors on the outcome of this patient cohort. Randomized controlled trials are needed to further evaluate the impact of SRS and possible combination approaches with surgery or WBRT as well as reliable prognostic factors on survival and tumor control in the future.

## 5. Conclusion

Stereotactic radiosurgery is a safe and effective treatment option in patients with brain metastases from RCC and results in excellent local control rates. In case of a limited number of brain metastases, surgery or SRS might be appropriate, depending on the individual characteristics of the patients and the number, size, and location of brain metastases. Further investigations such as randomized controlled trials are necessary for a reliable evaluation of prognostic factors and for a comparison of the outcome of patients treated with SRS alone versus combined treatment approaches.

## Figures and Tables

**Table 1:** Patient and disease characteristics by treatment group.

Characteristics	SRS	Surgery + SRS	WBRT + SRS	*p* value
Number of patients	36	24	6	
Age (years)				
Median age	60.5	58	59	0.5622
Mean age	58.6	58.1	62.8	
Range	31–79	40–85	54–81	
Sex				
Male	37 (75%)	19 (79.2%)	1 (16.7%)	0.909
Female	9 (25%)	5 (20.8%)	5 (83.3%)	
Systemic disease status at the time of SRS				
Controlled	3 (8.3%)	6 (25%)	1 (16.7%)	0.169
Uncontrolled	33 (91.7%)	18 (75%)	5 (83.3%)	
Intracranial disease status at the time of SRS				
Controlled	0 (0%)	12 (50%)	1 (16.7%)	<0.0001
Uncontrolled	36 (100%)	12 (50%)	5 (83.3%)	
RPA				
I	0 (0%)	3 (12.5%)	0 (0%)	0.1379
II	34 (94.4%)	20 (83.3%)	5 (83.3%)	
III	2 (5.6%)	1 (4.2%)	1 (16.7%)	
KPS				
≥70	34 (94.4%)	23 (95.8%)	5 (83.3%)	0.521
<70	2 (5.6%)	1 (4.2%)	1 (16.7%)	
SIR				
≥6	20 (55.6%)	11 (45.8%)	0 (0%)	0.041
<6	16 (44.4%)	13 (54.2%)	6 (100%)	
Ds-GPA				
4	18 (50%)	9 (37.5%)	0 (0%)	0.0073
3	8 (22.2%)	9 (37.5%)	1 (16.7%)	
2	8 (22.2%)	4 (16.7%)	2 (33.3%)	
1	2 (5.6%)	2 (8.3%)	2 (33.3%)	
0	0 (0%)	0 (0%)	1 (16.7%)	
BSBM				
3	0 (0%)	2 (8.3%)	0 (0%)	0.6526
2	3 (8.3%)	4 (16.7%)	1 (16.7%)	
1	28 (77.8%)	13 (64.2%)	3 (50%)	
0	5 (13.9%)	5 (20.8%)	2 (33.3%)	
Number of brain metastases				
≤3	33 (91.7%)	23 (95.8%)	2 (33.3%)	0.002
>3	3 (8.7%)	1 (4.2%)	4 (66.7%)	
Initial tumor volume (cm^3^)				
Median initial tumor volume	1.151	11.746	1.945	0.0001
Mean initial tumor volume	3.3770	11.411	9.295	
Range	0.241–27.73	2.178–26.51	0.192–33.57	

Fisher and Kruskal-Wallis test.

**Table 2:** Treatment characteristics.

Characteristics	*n* (%)
Stereotactic radiosurgery	
Median tumor volume (cm^3^)	0.688
Range tumor volume (cm^3^)	0.056–33.57
Median number of beams	202
Median number of monitor units	14741.26
Median dose per fraction (Gy)	22
Range dose per fraction (Gy)	5–22
Median total dose (Gy)	22
Range total dose (Gy)	12–30
Median number of fractions	1
Range number of fractions	1–5
Median coverage (%)	96.39
Median isodose line (%)	76
Range isodose line (%)	58–89
Median conformality index	1.3
Median homogeneity index	1.32
Median minimum dose (Gy)	2037.18
Median maximum dose (Gy)	2822.02
Surgical resection	
*N* (patients)	24
Gross total resection	22 (91.7)
Subtotal resection	2 (8.3)
Whole-brain radiation therapy (WBRT)	
*N* total	12 (18.2)
*N* WBRT prior to SRS	6 (9.1)
*N* WBRT after SRS	6 (9.1)
Median total dose (Gy)	30
Dose range (Gy)	20–37.5
Median dose for WBRT prior to SRS (Gy)	30
Dose range for WBRT prior to SRS (Gy)	20–30
Systemic therapy	
*N* (patients)	56 (84.8)
Standard	25 (44.6)
Experimental	5 (9)
Both	26 (46.4)

**Table 3:** Prognostic factors.

Variable	Statistical analysis			
	Log-rank	Multivariate Cox regression		
	*p* value	*p* value	Coefficient (Coeff.)	95% Confidence interval (CI)
Survival				
Age	0.0000	0.038	0.0338118	0.0018982–0.0657254
Prior surgery	0.0486	0.053	0.6203678	−1.24931–0.0085739
Prior WBRT	0.0097	0.014	1.104396	0.2219025–1.986891
RPA	0.0000	0.000	2.153972	1.108619–3.199326
KPS (≥70 versus <70)	0.0000	0.000	2.072708	0.9231157–3.222301
SIR (≥6 versus <6)	0.0093	0.011	2.155395	0.1726184–1.36333
BSBM	0.0027	0.003	−0.8503598	−1.402409–−0.2983109
Number of brain metastases (>3 versus ≤3)	0.0009	0.002	1.260513	0.462241–2.058785
Ds-GPA	0.0002	0.000	0.5929137	−0.7985645–−0.2468485
Initial tumor volume	0.0000	0.651	0.008675	−0.0289586–0.0463086

Local control				
Tumor volume	0.0000	0.668	−0.0242895	−0.1353437–0.0867646

Distant brain progression-free survival				
Prior WBRT	0.0072	0.014	1.252507	0.2539636–2.25105

**Table 4:** Summary of literature on SRS for renal cell brain metastasis.

Study	Year	Number of patients	Number of lesions	Median tumor volume (cm^3^)	Dose range (Gy)	Radiosurgery device	Median overall survival (months)	One-year local control (%)	One-year distant progression-free survival (%)
Present study	2014	66	207	0.688	12–30	Cyberknife	13.9	84% (SRS only) 94% (Surgery + SRS) 88% (WBRT + SRS)	35.2

Seastone et al. [31]	2014	166	487	1.96	12–35	Gamma knife	ND	90^a^	ND

Lwu et al. [32]	2013	16	41	0.4^f^	15–25	Gamma knife	ND	91	ND

Kim et al. [9, 14]	2012	46	99	3.0^b^	12–25	Gamma knife	10	84.7^a^	ND

Kano et al. [29]	2011	158	531	2.8	10–22	Gamma knife	8.2	86	45

Nieder et al. [43]	2011	35	ND	ND	ND	ND	10.1	ND	ND

Lo et al. [33]	2011	14	22	4^b,f^	15–22^f^	Gamma knife	6.5^f^	95.5^a^	40.2^f^

Fokas et al. [24]	2010	51	ND	ND	15–22	LINAC	12	81	ND

Marko et al. [34]	2010	19	59	1.72^b^	21.3^b^	Gamma knife	12.58	95^a^	ND

Hara et al. [44]	2009	18	145^f^	1.47^f^	14–24^f^	Cyberknife	14.2	87^f^	38^f^

Shuch et al. [45]	2008	138	ND	1.7	ND	ND	10.7^j^	ND	ND

Powell et al. [35]	2008	23	303^g^	ND	8–30^g^	Gamma knife	5.1^g^	93.6	37.3^g^

Jensen et al. [21]	2008	28	59	0.9	15–22	LINAC	7.03^f^	60.9^a^	ND

Samlowski et al. [36]	2008	32	71	0.03–26.9^d^	15–24	LINAC	6.7	86	ND

Shuto et al. [30]	2006	69	314	1.5^b^	8–30	Gamma knife	9.5	82.6^a^	ND

Manon et al. [46]	2005	14	ND	ND	15–24^g^	ND	8.3^g^	67.8^g,h^	67.8^g,h^

Chang et al. [16]	2005	77	99	1.5	15–24	LINAC	9.1	64.3	60

Muacevic et al. [37]	2004	85	376	1.2	15–35	Gamma knife	11.1	94^a^	ND

Noel et al. [19]	2004	28	65	1.28	10.9–22.3	LINAC	11	93	70

Sheehan et al. [7]	2003	69	146	2.8	12.5–32	Gamma knife	6	96^a^	ND

Petrovich et al. [47]	2002	29	70	ND	20^c^	Gamma knife	12	ND	ND

Hernandez et al. [17]	2002	29	92	4.7	13–30	Gamma knife	7	ND	ND

Siebels et al. [38]	2002	58	277	3.4	15–35	Gamma knife	9.9	95^a^	ND

Wowra et al. [39]	2002	75	350	1.6	15–35	Gamma knife	11	95^i^	ND

Hoshi et al. [18]	2002	42	110	1.5^c,e^	20–30	Gamma knife	12.5	93^a^	ND

Gerosa et al. [40]	2002	74	102	ND	22^b^	Gamma knife	14.6	86^a^	ND

Brown et al. [15]	2002	16	ND	ND	12–25	Gamma knife	17.8	85^a^	ND

Amendola et al. [20]	2000	22	ND	3.9^b^	15–22	Gamma knife	8	98.5^a^	ND

Payne et al. [41]	2000	21	37	4.4	10.5–40	Gamma knife	8	100^a^	ND

Goyal et al. [42]	2000	29	66	1.135	7–24	LINAC and Gamma knife	6.7	91^a^	ND

Schöggl et al. [8]	1998	23	44	ND	8–30	Gamma knife	11	96^a^	ND

Mori et al. [28]	1998	35	52	2.4^b^	13–20	Gamma knife	11	90^a^	ND

ND: not defined, LINAC: linear accelerator.

^a^Crude.

^b^Mean.

^c^Median.

^d^Range.

^e^mm tumor diameter.

^f^For melanoma and RCC.

^g^For melanoma, RCC, and sarcoma.

^h^At 6 months.

^i^At 1.5 years.

^j^From the time of diagnosis of the first brain metastasis.
